# Development and validation of a long-read metabarcoding platform for the detection of filarial worm pathogens of animals and humans

**DOI:** 10.1186/s12866-023-03159-3

**Published:** 2024-01-20

**Authors:** Lucas G. Huggins, Ushani Atapattu, Neil D. Young, Rebecca J. Traub, Vito Colella

**Affiliations:** Melbourne Veterinary School, Faculty of Science, University of Melbourne, Parkville, VIC 3050 Australia

**Keywords:** Next-generation sequencing (NGS), Nanopore, MinION™, Filarioid, *Dirofilaria*, *Brugia*, *Wuchereria*, Nemabiome, Vector-borne Disease (VBD)

## Abstract

**Background:**

Filarial worms are important vector-borne pathogens of a large range of animal hosts, including humans, and are responsible for numerous debilitating neglected tropical diseases such as, lymphatic filariasis caused by *Wuchereria bancrofti* and *Brugia* spp., as well as loiasis caused by *Loa loa*. Moreover, some emerging or difficult-to-eliminate filarioid pathogens are zoonotic using animals like canines as reservoir hosts, for example *Dirofilaria* sp. ‘hongkongensis’. Diagnosis of filariasis through commonly available methods, like microscopy, can be challenging as microfilaremia may wane below the limit of detection. In contrast, conventional PCR methods are more sensitive and specific but may show limited ability to detect coinfections as well as emerging and/or novel pathogens. Use of deep-sequencing technologies obviate these challenges, providing sensitive detection of entire parasite communities, whilst also being better suited for the characterisation of rare or novel pathogens. Therefore, we developed a novel long-read metabarcoding assay for deep-sequencing the filarial nematode cytochrome c oxidase subunit I gene on Oxford Nanopore Technologies’ (ONT) MinION™ sequencer. We assessed the overall performance of our assay using kappa statistics to compare it to commonly used diagnostic methods for filarial worm detection, such as conventional PCR (cPCR) with Sanger sequencing and the microscopy-based modified Knott’s test (MKT).

**Results:**

We confirmed our metabarcoding assay can characterise filarial parasites from a diverse range of genera, including, *Breinlia*, *Brugia*, *Cercopithifilaria*, *Dipetalonema*, *Dirofilaria*, *Onchocerca*, *Setaria*, *Stephanofilaria* and *Wuchereria*. We demonstrated proof-of-concept for this assay by using blood samples from Sri Lankan dogs, whereby we identified infections with the filarioids *Acanthocheilonema reconditum*, *Brugia* sp. Sri Lanka genotype and zoonotic *Dirofilaria* sp. ‘hongkongensis’. When compared to traditionally used diagnostics, such as the MKT and cPCR with Sanger sequencing, we identified an additional filarioid species and over 15% more mono- and coinfections.

**Conclusions:**

Our developed metabarcoding assay may show broad applicability for the metabarcoding and diagnosis of the full spectrum of filarioids from a wide range of animal hosts, including mammals and vectors, whilst the utilisation of ONT’ small and portable MinION™ means that such methods could be deployed for field use.

**Supplementary Information:**

The online version contains supplementary material available at 10.1186/s12866-023-03159-3.

## Background

Filarial worms, i.e., species of the superfamily Filarioidea, generate a significant pathogenic burden on numerous mammalian species, including humans [1–4]. Canines are also afflicted by many species of filarioids, e.g., those within the genera *Acanthocheilonema*, *Brugia*, *Dirofilaria* and *Onchocerca*, and can act as important reservoir hosts for zoonotic filarial worm species, thereby playing a key role in the maintenance of their transmission to humans [5–8]. For example, *Brugia* species invade the lymphatic system and although both *Brugia pahangi* and *Brugia malayi* do not cause severe disease in naturally infected dogs, the latter species is responsible for about 10% of human lymphatic filariasis cases worldwide [9, 10]. Dogs may also act as reservoir hosts for *Dirofilaria repens* which can cause ocular dirofilariasis in people [7, 8] and *D. immitis* which is occasionally found causing respiratory disease in humans, as well as in rare occasions generating ocular complications and secondary myocarditis [1, 11, 12]. More recently, the discovery in 2012 of *Dirofilaria* sp. ‘hongkongensis’ (*syn* ‘*Candidatus* Dirofilaria ‘hongkongensis’ and *Dirofilaria* sp. Hong Kong genotype), presents another canine-infecting filarioid that is also zoonotic, with this species found infecting both dogs and humans in Hong Kong, India, and Sri Lanka [6, 13–16]. In addition, the canine filarioid *Onchocerca lupi* is also a zoonotic species which may localise in the cervical spine of humans, particularly children, causing neurological complications and for which adulticidal treatment in dogs is not yet available [17–19].

Accurate diagnosis of filarial worm infections is challenging, particularly when using microscopic methods as morphological identification can be difficult between closely related species. Moreover, microfilaremia for some filarioids is periodic meaning that microfilaria only appear in the blood at certain times in the day, whilst at other points they may fluctuate down to diagnostically undetectable levels [20–22]. Concentration techniques such as the modified Knott’s test can increase the sensitivity of diagnosis by microscopy, although the challenges of morphological identification remain [20]. Serodiagnostic methods have also been widely utilised in the context of filarial worm diagnosis, nonetheless such methods may not be able to distinguish between active infections and historical ones, or have poor specificity and an inability to provide an exact, species-level diagnosis [22–24].

Traditional molecular diagnostic methods, such as conventional PCR (cPCR) and quantitative real-time PCR (qPCR), have been commonly used in filarial worm epidemiology and diagnosis, however these tools can typically only target one or a few species simultaneously, and in the case of cPCR may struggle to diagnose coinfections [23, 25–29]. Because of these limitations, novel technologies such as next-generation sequencing (NGS) have come to the fore, whereby species diagnostic barcoding genes can be amplified and sequenced to generate a ‘metabarcode’ of all the pathogens infecting a host [30–33]. In the field of parasitic nematode research such methods have been used to characterise the gastrointestinal ‘nemabiome’, using deep-sequencing technology like the Illumina-platform to target the internal transcribed spacer 2 (ITS2) region and detect all Clade V nematodes [30, 34–37]. Whilst studies reporting experimental exploration of the gut nemabiome have been increasing, to date there have been no equivalent studies exploring the equivalent nemabiome in blood.

Advantageously, Oxford Nanopore Technologies (ONT) have developed portable sequencing equipment, such as their MinION™ device, that can sequence read lengths much greater than those of short-read NGS platforms [38–40]. In the context of metabarcoding such read lengths can provide much better taxonomic designation, e.g., sequencing of the full-length bacterial 16S ribosomal RNA gene or near full length apicomplexan 18S ribosomal RNA gene [41–44]. In the case of filarial worms, the almost full-length cytochrome c oxidase subunit 1 (COI) gene can easily be sequenced on the MinION™, confidently providing species-level classification due to interspecific genetic diversity at this locus [45–48].

Taking this into account, we set out to develop a filarial worm COI gene metabarcoding assay using ONT’ portable MinION™ sequencer. The overarching aim of developing such an assay is that it could be used to facilitate explorative epidemiological surveys of filarioid infections in humans, wild and domestic animals as well as for pathogen vector discovery in a manner independent of complex and bulky laboratory infrastructure. To optimise our method and demonstrate proof-of-concept, we benchmarked our novel assay on 100 canine blood samples from Sri Lanka that had previously been confirmed positive to zoonotic filarial worm infections, using conventional methods for their diagnosis.

## Methods

### Sampling and DNA extraction

To ensure that a wide range of filarial worm pathogens of veterinary and human health importance could be detected by our novel method, a diverse spectrum of positive control samples were sourced and tested (Table 1). These samples had previously been characterised using either conventional PCR and Sanger sequencing and/or microscopy and were typically canine blood extracted DNA or DNA extracted from filarial worms or their vectors (Table 1). These filarial worm positive control samples used for the development and validation of our metabarcoding assay had their DNA extracted using the DNeasy Blood and Tissue Kits (Qiagen, Hilden, Germany) according to the manufacturer’s protocol, with DNA eluted in 200 µl of buffer AE.

Additionally, the filarial metabarcoding assay was tested using 100 whole blood extracted DNA samples collected from locally owned dogs from eight veterinary clinics across Sri Lanka. These DNA extracts were a subset of samples from a previous study by Atapattu et al. (2023) which contains detailed information on sampling locations, the health of canines from these areas and the sample collection procedure. Collected blood samples were stored at -20 °C until they could be transported, frozen to the University of Peradeniya, Sri Lanka for DNA extraction. Extraction of whole blood was conducted in the same way as filarioid positive control sample.

All extracted DNA was kept at -20 °C until use. All DNA extracts were quantified using a Qubit™ 4 Fluorometer (Thermo Fisher Scientific, Massachusetts, USA) using the dsDNA HS assay kit.

### Filarial worm COI gene metabarcoding assay

For nanopore deep-sequencing experiments, DNA samples were shipped to the University of Melbourne, Australia at 4 °C. Library preparation for metabarcoding of the filarial worm COI gene on the MinION Mk1B sequencer (Oxford Nanopore Technologies, Oxford, UK) was conducted using both the PCR Barcoding Expansion 1–12 (EXP-PBC001) and the PCR Barcoding Expansion 1–96 (EXP-PBC096) with ONT’ Ligation Sequencing Kit (SQK-LSK110). Protocols followed were ‘Ligation sequencing amplicons - PCR barcoding (SQK-LSK110 with EXP-PBC001)’ version: PBAC12_9112_v110_revJ_10Nov2020 and ‘Ligation sequencing amplicons - PCR barcoding (SQK-LSK110 with EXP-PBC096)’ version: PBAC96_9114_v110_revK_10Nov2020, both with some modifications to improve yield. For the first step PCR amplification 25 µl PCRs were conducted using 12.5 µl of LongAmp® Hot Start Taq 2× Master Mix (New England Biolabs, Massachusetts, USA) 7.5 µl Ambion Nuclease-Free Water (Life Technologies, California, USA), 1 µl of forward primer Fil_COIint_ONT_F, 1 µl of reverse primer Fil_COIint_ONT_R and 3 µl of genomic blood extracted DNA from dogs. We used modified pan-filarial primers COIintF and COIintR described by Casiraghi et al. (2001) that amplify an approximately 650 base pair (bp) stretch of the filarial worm COI gene [48]. Modifications to these primers involved the addition of ONT adapter sequences (underlined) that permit the addition of DNA barcodes in a subsequent secondary PCR reaction, hence the primer sequences were Fil_COIint_ONT_F: 5’- TTTCTGTTGGTGCTGATATTGCTGATTGGTGGTTTTGGTAA − 3’ and Fil_COIint_ONT_R: 5’ – ACTTGCCTGTCGCTCTATCTTCATAAGTACGAGTATCAATATC − 3’. PCRs were then conducted on a T100™ Thermal Cycler (Bio-Rad, California, USA) using the following conditions: 1 cycle of 94 °C for 1 min, 25 cycles of 94 °C for 1 min, 50 °C for 45 s and 65 °C for 45 s, with a final extension of 65 °C for 10 min. Separate and different physical laboratory areas were utilised for DNA extraction, pre-PCR and post-PCR experiments with all first-step PCRs prepared in a PCR hood under sterile conditions with filter tips, following UV sterilisation of the workspace.

PCR product was then added to a 96-well plate and cleaned using a 1× ratio of NucleoMag NGS Clean-up and Size Select Beads (Macherey-Nagel, Duren, Germany) with a 15 min incubation on a HulaMixer (Thermo Fisher Scientific) and two washes with freshly made 75% ethanol, followed by a final elution in 25 µl of Ambion Nuclease-Free Water. Next second step PCRs were conducted to add ONT barcodes to each samples’ amplicon and thereby permit multiplexing of up to 96 samples onto a flow cell. These secondary PCRs were 50 µl reactions utilising 25 µl of LongAmp® Taq 2× Master Mix (New England Biolabs), 24 µl of cleaned PCR product from the first PCR reaction and 1 µl of a unique barcode from the ONT PCR Barcoding Expansion 1–96 kit. Thermocycling conditions for this second reaction were 1 cycle of 95 °C for 3 min, 12 cycles of 95 °C for 15 s, 62 °C for 15 s and 65 °C for 45 s, with a final extension of 65 °C for 5 min. After the second PCR, the PCR products underwent another clean-up step using a 0.7× ratio of NucleoMag beads to exclude and remove low weight (< 200 bp) PCR product, hence 35 µl of beads were used to clean 50 µl of PCR product with the same incubation and ethanol wash steps used as previously described and an elution in 20 µl of Ambion Nuclease-Free Water. Next correct amplification of the expected product was assessed using a subset of samples on a 4200 TapeStation System (Agilent Technologies, California, USA) and the final DNA concentrations of this subset analysed using a Qubit™ 4 Fluorometer. Subsequently, 3 µl of each barcoded and cleaned PCR product were pooled together (288 µl total pool for the batch of 96 samples and 30 µl total pool for the batch of ten) and concentrated down using a 2× ratio of NucleoMag beads, washed and eluted in 55 µl of Ambion Nuclease-Free Water. Amplicon pools were then quantified on a Qubit™ 4 Fluorometer to ensure there was adequate DNA (minimum of 1,000 ng) to be taken forward.

Final preparatory steps including DNA repair and end-prep, adapter ligation and clean-up and MinION™ flow cell priming and loading were conducted exactly as described in the relevant ONT’ protocols, making use of the NEBNext® Companion Module for Oxford Nanopore Technologies® Ligation Sequencing (New England Biolabs) and the ONT’ Ligation Sequencing Kit (SQK-LSK110). Final concentrations of sequencing library were always between 25 and 70 fmol.

For method development and optimisation samples were run in batches of 12, whilst for methodological comparison of our metabarcoding assay using the 100 canine blood samples from Sri Lanka, samples were run as a batch of 96 and a batch of ten. Batches for methodological comparisons were run with two no template PCR negative controls, i.e., nuclease free water, and four positive controls that were comprised of a uniquely identifiable 690 bp gBlock synthetic DNA strand (Integrated DNA Technologies, Iowa, USA) of the 16S rRNA gene from *Aliivibrio fischeri*, as previously reported by Huggins et al. [49]. This positive control gBlock consisted of the relevant 16S rRNA gene sequence flanked by the appropriate primer binding regions for the COIintF and COIintR primers as an artificial construct, the design of which, can be seen in Additional Information 1. The batch of 96 multiplexed amplicons was run on a new R9.4.1 flow cell (Oxford Nanopore Technologies), whilst smaller batches for diagnostic benchmarking or testing of positive controls were run on new or re-used R9.4.1 flow cells. If flow cells were re-used this was always after a DNAse clean-up using the EXP-WSH004 Flow Cell Wash Kit (Oxford Nanopore Technologies), to reduce the possibility of DNA contamination and carry-over from prior sequencing runs.

Nanopore sequencing was conducted on a MinION Mk1B device using a Legion 7i Gen 6 laptop (Lenovo, Quarry Bay, Hong Kong) that utilises a NVIDIA® GeForce RTX 3070 (8 GB) GPU and 11th Gen Intel® Core™ i7-11800 H (8 C) processor to permit field-based base-calling. Sequencing was initiated through MinKNOW version 22.05.5 with fast base-calling and a Q-score of ≥ 8, for between 5 and 25 h depending on the amount of data required. Once sequencing was stopped, FAST5 reads were base-called using the super high accuracy base-calling model with barcode removal using Guppy version 6.1.5. Upon sequencing commencement, the success of the sequencing run was assessed using MinKNOW to ensure reads were of the expected size and pore activity was healthy. Sequencing that was conducted for methodological comparison was allowed to continue until a mean raw read count of at least 190,000 reads was achieved per sample, although actual sample read counts varied substantially.

### Bioinformatics

Because ONT’ R9.4.1 flow cells have a relatively high error rate [50] when compared to other next-generation sequencing platforms, we used the bioinformatic pipeline NanoCLUST [51] that is capable of compensating for this error rate by generating highly accurate amplicon consensus sequences. This bioinformatic pipeline was chosen as it had previously shown great utility in forming 16S rRNA and 18S rRNA gene consensus sequences for bacterial and apicomplexan microbiome experiments at an accuracy as high as 99.7–100% identity to representative sequences from GenBank [43, 44, 51]. The NanoCLUST pipeline carries out multiple quality control, read clustering, polishing and consensus forming steps followed by classification of consensus sequences against a database of the user’s choice.

We generated a bespoke database that included all NCBI’s GenBank COI gene sequences > 100 and < 100,000 bp in length for the Filarioideae superfamily (taxid 6295). Data was downloaded using the search terms: (((((((((((cytochrome c oxidase subunit 1[Title]) OR cytochrome c oxidase subunit I) OR cytochrome oxidase subunit 1) OR cytochrome oxidase subunit I) OR COX1) OR CO1) OR COI)) AND txid6295[Organism:exp])) AND 100:100000[Sequence Length]). To this database we also included our positive control sequence for the *A. fischeri* 16S rRNA gene (NCBI accession NR_029255.1) and the *Canis lupus familiaris* genome (NCBI accession GCF_014441545.1) to permit classification of host sequences. The relevant GitHub page detailing how our database was constructed is available here https://github.com/vetscience/Huggins_NanoCLUST, our database is downloadable from https://melbourne.figshare.com/projects/Huggins_NanoCLUSTdb/160631.

Optimal NanoCLUST parameters were found to be to be a minimum read length of 490 bp, maximum read length of 1,700 bp, minimum cluster size of 50 and 100 reads used for polishing, with all other parameters as the pipeline’s defaults. Read counts as well as consensus sequence lengths and classifications generated by NanoCLUST were taken as the final dataset generated by our metabarcoding assay to which other diagnostic methods were compared. All metabarcoding NGS data produced in the present study are available from the NCBI BioProject database BioProjectID: PRJNA923029; BioSampleIDs SAMN32683217 to SAMN32683322, specifically Sequence Read Archive (SRA) accessions SRX19025169 to SRX19025274.

Importantly, for results generated by our metabarcoding assay read count thresholds had to be identified and employed to determine whether a sample was positive to a given filarial worm pathogen. The method for determining read thresholds for a given sequencing run batch were calculated as per Huggins et al. (2021b). In brief, reads of the uniquely identifiable *A. fischeri* positive control sequence that were found in samples other than the positive control were used to inform the read cut-off threshold for a given library. Identification of these positive control sequences in non-positive control samples is possible due to infrequent barcode index misreading, sequencing error, chimeric reads, or small amounts of cross-contamination during NGS library preparation [52, 53]. Positive controls were used as a pure sequence construct concentrate at a concentration of 10.3 × 10^− 5^ ng/µl, that produced a post-PCR DNA concentration substantially higher than that achieved by biological sample, making these sequences pose the greatest risk of generating index misreading or cross-contamination. Therefore, to be able to take the strictest possible read cut-off threshold for a given sequencing batch, the cut-off was taken to be the highest read-count of a positive control sequence, i.e. *A. fischeri* sequence, in a non-positive control sample. If filarial worm reads from a blood sample were found to be lower than the batch’s threshold then the sample was defined as negative for that pathogen, i.e., a false positive.

### Diagnostic test methodological comparisons

The 100 Sri Lankan canine blood samples that were analysed through our metabarcoding assay were also assessed using two additional diagnostic methods, the modified Knott’s test (MKT) and conventional PCR (cPCR) targeting the COI gene [48] with Sanger sequencing. The Knott’s test was carried out as described by Atapattu et al. (2023) with morphological features and measurements used to characterise microfilariae observed as belonging to either the genus *Brugia* spp. or *Dirofilaria* spp.

For cPCR and Sanger sequencing analysis canine blood extracted DNA underwent PCR using COIintF and COIintR primers [48] in 20 µl reactions as described by Atapattu et al. (2023). PCR product was visualised on 1.5% agarose gels using a ChemiDoc™ Imaging System (Bio-Rad, California, USA) with samples found positive by cPCR purified and sent to Macrogen (Seoul, South Korea) for Sanger sequencing. Samples that returned a utilisable Sanger sequencing chromatogram were edited in Geneious Prime® version 2022.2.1 (Geneious, New Zealand) and classified using BLASTn in NCBI’s GenBank.

Kappa statistics were used to compare diagnostic test agreement of the metabarcoding assay results against those achieved by MKT and cPCR with Sanger sequencing by Atapattu et al. (2023) for the pathogens *Brugia* sp. Sri Lanka (SL) genotype and *Dirofilaria* sp. ‘hongkongensis’. Kappa statistics were conducted using SPSS Statistics 28 (IBM, New York, USA), employing the formula defined by McHugh (2012) for categorising levels of agreement between two tests, i.e., inter-test agreement was considered poor if k ≤ 0.20, fair if 0.21 ≤ k ≤ 0.40, moderate if 0.41 ≤ k ≤ 0.60, substantial if 0.61 ≤ k ≤ 0.80, and high if k > 0.81 [54].

## Results

### Methodological validation and optimisation of filarial worm metabarcoding assay

The developed metabarcoding method was able to accurately characterise a diverse range of validated positive controls for different filarial worm pathogens obtained from a variety of sample types and animal hosts. This method amplified and formed consensus sequences with over 99.6% identity to the relevant representative sequences from GenBank, except for *Dipetalonema gracile* at 98.7% identity (Table 1). Accurate results were also obtained in the context of coinfections with two closely related filarial worm species, e.g., *D. repens* and *D. immitis*, whereby generation of both COI consensus sequences was achieved (Table 1). Additionally, accurate results were attainable from whole blood extracted DNA samples with low total DNA concentrations (< 0.1 ng/µl) as was the case for the Whatman FTA card blood spot extracted DNA positive to *Wuchereria bancrofti* (Table 1). Bioinformatic processing with NanoCLUST generated highly accurate filarial worm COI gene consensus sequences and did not overinflate nor underestimate the filarial worm diversity of controls.

During the NucleoMag bead clean-up steps for the product of the second PCR a ratio of 0.7× beads was used for size selection which removed all reads of length 200 bp or less and increased the overall run output for COI gene sequences. In addition, although it is typically recommended that equimolar quantities of indexed amplicons are pooled together, we found that this was unachievable given the large disparities in amplicon DNA concentrations attained after our secondary PCR reaction when using the Sri Lankan dog blood samples (< 0.13 ng/µl to > 100 ng/µl). Instead, pooling of 3 µl of secondary PCR product from all samples and controls, followed by concentration of this pool with a 2× NucleoMag bead ratio within ~ 47 µl of water eluent could still generate an NGS library with every multiplexed sample represented. After the DNA repair, end-prep and adapter ligation steps, final loading concentrations were occasionally above the 5–50 fmol recommended for R9.4.1 flow cells. Nonetheless, with final concentrations as high as ~ 70 fmol, sequencing was successful, providing sequencing runs that retained pore activity and high output even after 43 h of sequencing time.

**Table 1:** Performance of our COI gene-targeting metabarcoding assay and NanoCLUST pipeline classification on reference filarial worm controls

Filarial worm pathogen(s) present	Sample type	NanoCLUST classification	NCBI accession no.	Length (bp)	Identity (%)	No. of reads
*Acanthocheilonema reconditum*	Canine blood	*Acanthocheilonema reconditum*	JF461456.1	496	99.6	22,241
*Breinlia boltoni*	Whole adult worm from possum lung	*Breinlia boltoni*	OP040125.1	650	100	46,652
*Brugia* sp. Sri Lanka (SL) genotype	Canine blood	*Brugia* sp. Sri Lanka (SL) genotype	MN564741.1	630	99.7	99,070
*Brugia* sp. SL genotype and *Dirofilaria* sp. ‘hongkongensis’	Canine blood	*Brugia* sp. SL genotype, *Dirofilaria* sp. ‘hongkongensis’	MN564741.1, KX265050.1	664, 679	99.7, 99.9	2172; 76,144
*Cercopithifilaria rugosicauda*	Tick haemolymph	*Cercopithifilaria rugosicauda*	KC610815.1	514	99.8	88,207
*Dipetalonema gracile*	Whole worm from monkey abdominal cavity	*Dipetalonema gracile*	KP760179.1	628	98.7	75,145
*Dirofilaria immitis*	Canine blood	*Dirofilaria immitis*	MW577348.1	688	99.7	5695
*Dirofilaria repens* and *Dirofilaria immitis*	Canine blood	*Dirofilaria repens, Dirofilaria immitis*	MW675691.1, MW577348.1	668, 687	99.9, 99.6	7923; 69,844
*Dirofilaria* sp. ‘hongkongensis’	Canine blood	*Dirofilaria* sp. ‘hongkongensis’	KX265050.1	600	99.8	89,455
*Onchocerca gibsoni* and *Stephanofilaria* sp.	Lesion swab from cow	*Onchocerca gibsoni, Stephanofilaria* sp.	AJ271616.1, MW143322.1	649, 425	99, 100	218; 675
*Onchocerca lupi*	Clinical sample, whole adult worm from canine eye	*Onchocerca lupi*	JX080029.1	686	100	72,533
*Setaria tundra*	Mosquito haemolymph	*Setaria tundra*	KF692103.1	676	99.9	66,504
*Wuchereria bancrofti*	Whatman FTA card blood spot from human	*Wuchereria bancrofti*	AP017705.1	684	99.4	78,220

Classification information shows the top hit(s), identity, and length of the relevant filarial worm consensus sequence as generated by the NanoCLUST pipeline, when classified using BLASTn on our bespoke filarial worm COI gene database. Note that relevant NCBI accession numbers were taken from GenBank as they are not automatically outputted by NanoCLUST

### Filarial worm metabarcoding of 100 Sri Lankan dog blood DNA samples

From sequencing batches used to conduct filarial worm metabarcoding of 100 canine blood samples and six control samples (four positive and two negative), a total of 20,419,427 raw reads (352.21GB total data, i.e., FAST5 and FASTQ) were generated that were processed and filtered by NanoCLUST into 3,908,026 polished and utilisable reads. The ratio of passed bases (those that met a Q-score of ≥ 8) to failed bases, was also good at 14.4GB:2.4GB for the 96-sample sequencing batch (sequenced for 43 h) and 1.2GB:0.4GB for the final 10-sample sequencing batch (sequenced for 5.5 h). Pre- and post-filtering sample read counts varied substantially with the mean and S.E. across all biological samples pre-filtering being 176,733 ± 36,140 and post-filtering being 34,314 ± 10,183. Additionally, the total read count for positive controls pre-filtering was 2,746,110 with a mean count of 549,222 ± 143,362, whilst post-filtering total reads were 476,672 with a mean count of 95,334 ± 1,391 across all four positive controls. For negative controls pre-filtering, the total read count was 44 with a mean count of 15 ± 5, whilst zero reads made it through the NanoCLUST filtering stage. Read counts were not normally distributed hence the average count is better represented by the median which was 2,212 for read counts pre-filtering with a range of 6 to 1,765,401 and 2,129 for read counts post-filtering with a range of 0 to 997,775.

Utilising our NanoCLUST dataset collated from the relevant sequencing batches our filarial worm COI gene targeting assay detected the pathogens *Acanthocheilonema reconditum*, *Brugia sp.* SL genotype and *Dirofilaria* sp. ‘hongkongensis’ from the 100 Sri Lanka dog blood samples tested (Table 2). The only other sequencing hits returned were from our *A. fischeri* positive control sequence and from the host; *Canis lupus familiaris*, the latter sequences of which were omitted from the final dataset. Using our formula defined in the ‘Methods’ for determining read thresholds for filarial worm positivity, we found all cut-off thresholds to be at low-to-medium read counts of between 358 to 4,048 reads for all sequencing batches. Nonetheless, the relevant batch cut-off threshold was ignored for the pathogen *A. reconditum* as the very limited number of samples found positive to this filarial worm were deemed too low to represent putative index misreading or cross-contamination.

Overall, 58% of dogs tested were infected with at least one filarial worm pathogen, whilst coinfections were also common, with 16% of dogs found to be infected with two filarioid nematodes simultaneously (Table 2). For canines coinfected with two filarial worms, the proportion of species read counts varied with one species typically dominating and comprising the majority of reads, (most frequently *Dirofilaria* sp. ‘hongkongensis’) although one sample was balanced with almost equal read counts for both *Brugia* sp. SL genotype and *Dirofilaria* sp. ‘hongkongensis’.

**Table 2:** Results obtained by the metabarcoding assay on 100 dog blood DNA samples from Sri Lanka

Filarial worm(s) identified	No. of samples positive
**Single infections**	
*Acanthocheilonema reconditum*	1
*Brugia* sp. SL genotype	5
*Dirofilaria* sp. ‘hongkongensis’	36
**Total single infections**	42
**Coinfections**	
*Acanthocheilonema reconditum* and *Dirofilaria* sp. ‘hongkongensis’	2
*Brugia* sp. SL genotype and *Dirofilaria* sp. ‘hongkongensis’	14
**Total coinfections**	16
**Single infections and coinfections**	
*Acanthocheilonema reconditum*	3
*Brugia* sp. SL genotype	19
*Dirofilaria* sp. ‘hongkongensis’	52
**Total infected dogs**	58
**Total uninfected dogs**	42

Classifications were obtained using the NanoCLUST pipeline and BLASTn identification on our filarial worm COI gene database

### Diagnostic test methodological comparisons

Through test result comparisons on the same Sri Lankan dog samples, between the metabarcoding assay versus traditional tests that are commonly used for filarial worm diagnosis, we benchmarked our new method and compared its diagnostic performance. For the pathogens *Brugia* sp. SL genotype and *Dirofilaria* sp. ‘hongkongensis’ the results of the three tests could be directly compared using kappa agreement statistics. Table 3 shows the agreement statistics between both molecular methods compared; cPCR with Sanger sequencing and metabarcoding. Agreement between these two methods for detection of both comparable filarioids was moderate, i.e., a kappa value of 0.41 ≤ k ≤ 0.60, with discordance primarily driven by samples found positive for both filarioids by metabarcoding that were negative by cPCR. For example, of the total *Brugia* sp. SL genotype infections identified by both methods 58% were only detected by the metabarcoding method and for *Dirofilaria* sp. ‘hongkongensis’ 40% were only detected by metabarcoding. The metabarcoding assay identified more filarioid coinfections, finding 16 samples positive to a coinfection (Table 3), whilst cPCR with Sanger sequencing could not detect coinfections. Conventional PCR with Sanger sequencing was unable to detect any *A. reconditum* infections, with this pathogen solely detected using the metabarcoding assay (Table 2).

The results of our metabarcoding assay were also compared to those achieved by the MKT. Results were compared at the genus level as morphological characteristics of microfilaria alone cannot be used to provide species-level classification for some *Dirofilaria* species, e.g., for distinguishing *D. repens* from *Dirofilaria* sp. ‘hongkongensis’ [6]. Kappa statistics showed either poor agreement between results obtained for pathogens of the genus *Brugia* spp. or fair agreement for pathogens of the genus *Dirofilaria* spp. with discordance mainly driven by infections missed by the MKT but detected by the metabarcoding assay (Table 4). Of the total *Dirofilaria* spp. infections detected by both methods 64% were uniquely detected by metabarcoding, whilst in contrast the MKT uniquely identified just one infection, i.e., 2% (Table 4). Of the total *Brugia* spp. infections 89% were only detected by the metabarcoding assay. Furthermore, to provide greater clarity on the relative sensitivity of the three tests to detect filarial worm infections we compared the results of the MKT with those achieved by cPCR and Sanger sequencing (Table 5). Between these two tests kappa agreement was fair for detection of *Brugia* spp. and moderate for detection of *Dirofilaria* spp. with result disparities predominantly caused by filarial worm positive samples missed by the MKT and detected by cPCR with Sanger sequencing (Table 5). No *Acanthocheilonema* spp. microfilaria were detected for any Sri Lankan dog sample by the MKT, nor were any COI gene sequences obtained for this genus via cPCR with Sanger sequencing.

**Table 3:** Conventional PCR with Sanger sequencing versus metabarcoding assay agreement statistics for the two most common filarial worm pathogens identified from 100 Sri Lanka dog blood samples

Filarial worm	cPCR and Sanger sequencing	Metabarcoding assay		Total agreement (%)	Kappa (95% CI)	Agreement	Kappa SE
		NEG	POS				
*Brugia* sp. SL genotype	**NEG**	81	11	89	0.541 (0.425–0.657)	Moderate	< 0.001
	**POS**	0	8				
*Dirofilaria* sp. ‘hongkongensis’	**NEG**	47	21	78	0.566 (0.491–0.641)	Moderate	< 0.001
	**POS**	1	31				

POS = positive, NEG = negative, CI = confidence intervals, SE = standard error. Agreement level defined as poor if coefficient (k) is ≤ 0.20, fair agreement if 0.21 ≤ k ≤ 0.40, moderate agreement if 0.41 ≤ k ≤ 0.60, substantial agreement if 0.61 ≤ k ≤ 0.80, and high agreement if k > 0.81

**Table 4:** Modified Knott’s test versus metabarcoding assay agreement statistics for the two most common filarial worm pathogens identified from 100 Sri Lanka dog blood samples

Filarial worm	Modified Knott’s test	Metabarcoding assay		Total agreement (%)	Kappa (95% CI)	Agreement	Kappa SE
		NEG	POS				
*Brugia* spp.	**NEG**	81	17	83	0.16 (0.059–0.261)	Poor	0.003
	**POS**	0	2				
*Dirofilaria* spp.	**NEG**	47	34	65	0.317 (0.246–0.388)	Fair	< 0.001
	**POS**	1	18				

POS = positive, NEG = negative, CI = confidence intervals, SE = standard error. Agreement level defined as poor if coefficient (k) is ≤ 0.20, fair agreement if 0.21 ≤ k ≤ 0.40, moderate agreement if 0.41 ≤ k ≤ 0.60, substantial agreement if 0.61 ≤ k ≤ 0.80, and high agreement if k > 0.81. Genus-level taxonomic classifications are shown as the modified Knott’s test can only identify microfilaria to this taxonomic level, whilst our metabarcoding protocol could classify down to species level in all instances

**Table 5:** Modified Knott’s test versus cPCR and Sanger sequencing agreement statistics for the two most common filarial worm pathogens identified from 100 Sri Lanka dog blood samples

Filarial worm	Modified Knott’s test	cPCR and Sanger sequencing		Total agreement (%)	Kappa (95% CI)	Agreement	Kappa SE
		NEG	POS				
*Brugia* spp.	**NEG**	92	6	94	0.38 (0.187–0.573)	Fair	< 0.001
	**POS**	0	2				
*Dirofilaria* spp.	**NEG**	66	15	83	0.562 (0.472–0.652)	Moderate	< 0.001
	**POS**	2	17				

POS = positive, NEG = negative, CI = confidence intervals, SE = standard error. Agreement level defined as poor if coefficient (k) is ≤ 0.20, fair agreement if 0.21 ≤ k ≤ 0.40, moderate agreement if 0.41 ≤ k ≤ 0.60, substantial agreement if 0.61 ≤ k ≤ 0.80, and high agreement if k > 0.81. Genus-level taxonomic classifications are shown as the modified Knott’s test can only identify microfilaria to this taxonomic level, whilst cPCR and Sanger sequencing could classify down to species level in all instances

## Discussion

We have herein shown how long-read nanopore sequencing technology can be used to accurately detect and characterise a diverse range of filarial worms from mammals, including humans, and from arthropod vectors. Through targeted amplification and sequencing of the COI gene of filarioids the metabarcode can be elucidated, providing insight into the presence of mono- or coinfections in an assumption free manner capable of detecting rare, emerging, or even novel filarial worm pathogens that are commonly missed by traditional diagnostic approaches. Metabarcoding’s ability to detect all filarial worms from a host is of great value, potentially making such methods useful in the context of human filariasis elimination programs, for example in regions endemic for both *Brugia* spp. and *W. bancrofti*. Moreover, in contexts where *Onchocerca volvulus* and *Loa loa* are co-endemic, mass drug administration (MDA) with ivermectin for onchocerciasis may be unsafe as potentially lethal post-ivermectin encephalopathy can occur for individuals infected with *L. loa* at high microfilarial densities [55–57]. In these scenarios, human testing must occur prior to MDA with the use of sensitive and specific molecular methods like metabarcoding, on sample types such as blood or skin-snips, conferring the ability to accurately detect all filarioids.

The wide range of positive controls our metabarcoding assay was tested against demonstrates the possible utility of our method to be used within studies investigating the epidemiology of filarial worms from varied vectors and animal hosts, including humans. For example, DNA extracted from FTA Whatman card blood spots that had total DNA concentrations lower than 0.1 ng/µl could be amplified and sequenced. In this case, our metabarcoding assay correctly identified a sample collected from American Samoa as being positive to *W. bancrofti* [58], the principal agent of human lymphatic filariasis [46]. This data is important, highlighting the potential for our method to not only be used for epidemiological surveys of human lymphatic filariasis, but also because sampling using blood spot collection on Whatman cards is more simplistic and may be subject to less strict importation biosecurity, allowing such studies to be carried out more easily [22, 59]. Filarial worm species, such as *O. gibsoni* and *O. lupi* that have microfilaria that are released into the dermis [29, 60] were also tested. These species are not typically detectable using blood samples, however our demonstration of the metabarcoding assay’s ability to characterise these species is useful given as this method could show great utility as an arthropod vector bio-surveillance tool for filarioids. In addition, it is likely that the range of filarial worm species detectable is not limited to those tested in the present study, given that the herein employed COIintF and COIintR primers have been used to successfully amplify the COI gene from the following additional species; *Acanthocheilonema viteae*, *Brugia pahangi*, *Dipetalonema* spp. *Litomosoides sigmodontis*, *Loa loa*, *Mansonella ozzardi*, *Mansonella perstans*, *Onchocerca fasciata*, *Onchocerca gutturosa*, *Onchocerca ochengi*, *Onchocerca volvulus*, *Setaria digitata* and *Thelazia* spp [47, 48, 61–66].

Using our metabarcoding assay, COI gene consensus sequences obtained for a wide spectrum of filarioids were almost exclusively over 99.6% identical to representative sequences from GenBank, demonstrating a sequencing and bioinformatic processing accuracy easily capable of classifying pathogens down to a species-level. Such results demonstrate the capability of the NanoCLUST pipeline, to error correct and accommodate for ONT’ R9.4.1 flow cells raw read error rate of approximately 97%, permitting identification of coinfections caused by even closely related filarioids [51, 67]. The only filarial worm that generated a result through our bioinformatic pipeline less than 99.6% identical to the relevant reference sequence was for the species *D. gracile*. This may be explained by the fact that this filarioid is suspected of comprising a species complex with high levels of diversity across its mitochondrial genome [68]. Additionally, our metabarcoding assay demonstrated superior diagnostic performance when compared to commonly used methods for filarial worm diagnosis, detecting more infections, especially coinfections, than the MKT or cPCR with Sanger sequencing. The benefits of metabarcoding with respect to coinfection detection makes this method of great use for filarial worm discovery and elucidation of novel vectors, as arthropods may be coinfected with multiple filarioids simultaneously [69].

To evaluate the utility of our metabarcoding assay, particularly for detection of zoonotic filarial worms, we tested it on 100 Sri Lankan dog samples as a proof-of-concept. From these samples the filarioids *Acanthocheilonema reconditum*, *Brugia* sp. SL genotype and zoonotic *Dirofilaria* sp. ‘hongkongensis’ were detected. *Dirofilaria* sp. ‘hongkongensis’ has been identified from Sri Lanka and the southern Indian regions of Tamil Nadu and Kerala before and is a relatively novel filarial worm species that has an unknown pathogenicity in canine hosts [6, 14, 15, 70, 71]. Nonetheless, this species is of significant zoonotic concern as adult worms have been found in subcutaneous nodules from humans in India and Hong Kong, with infection associated with lymphadenopathy, ocular complications, as well as the risk of a severe anaphylactic reaction upon parasite excision [13, 14, 16, 71, 72]. Importantly, Sri Lanka has one of the highest rates of human subcutaneous dirofilariasis of any country globally [73]. The employment of metabarcoding and other molecular methods is of great importance as cryptic species such as *Dirofilaria* sp. ‘hongkongensis’ may go undetected when using microscopic methods due to them being morphologically difficult to distinguish from better characterised filarioids like *D. repens* [6, 70, 74]. Moreover, currently developed antigen tests for *D. repens* are unable to detect *Dirofilaria* sp. ‘hongkongensis’, meaning that dogs may act as an undetected reservoir if serodiagnostic methods are predominantly used to surveil for this species [15, 70].

The detection of *Brugia* sp. SL genotype by our metabarcoding assay is also important as it may generate a similar pathogenesis in humans to the closely related *B. malayi*, a filarioid that is responsible for approximately 10% of human lymphatic filariasis cases globally, with most human and animal cases found in South and Southeast Asia [9, 75]. When infecting people, *B. malayi* can cause severe lymphedema and lymphatic blockage resulting in elephantiasis, thereby causing substantial morbidity, disfigurement, and distress to infected individuals [76–78].

Our metabarcoding assay detected substantially more filarial worm infections than the traditional molecular diagnostic method we compared it against, identifying 11 more *Brugia* sp. SL genotype and 21 more *Dirofilaria* sp. ‘hongkongensis’ infections, i.e., of the total infections for these pathogens 58% and 40% respectively, were only found by metabarcoding. Such discrepancies were reflected in the kappa agreement statistics calculated between these two tests that showed moderate agreement, i.e., with a k value ≥ 0.41 and ≤ 0.6, for both filarioid pathogens. This is particularly surprising given that both of these molecular techniques used the same PCR primers. Nonetheless, similar studies have found comparable results, whereby metabarcoding using the Illumina platform identified more pathogen positive samples than cPCR with Sanger sequencing [32, 79, 80]. Such findings may represent a diagnostic limit of detection for cPCR as samples can only be Sanger sequenced successfully upon amplification and visualisation of a PCR product band, with such bands only observable above a certain threshold concentration of DNA [81–83]. This threshold concentration may be much lower or non-existent for deep sequencing methodologies which can successfully amplify from PCR product, even if no such product is visualisable on a gel [84]. In addition, our metabarcoding assay was the only method tested that could detect *A. reconditum*, re-identifying this pathogen in dogs from Sri Lanka for the first time since 1962 [85]. The lack of *A. reconditum* detection by traditional diagnostics could be due to this pathogen potentially releasing reduced numbers of microfilariae into the bloodstream, as supported by the absence of detection of this species via the MKT. Therefore, the amount of *A. reconditum* DNA within the blood may be below the limit of detection for cPCR with Sanger sequencing.

The improved ability of our metabarcoding assay to detect filarial worm poly-parasitism was also exhibited. Sixteen more coinfections were identified by our metabarcoding method when compared to cPCR with Sanger sequencing, highlighting a significant limitation of the latter technique, i.e., that this method can typically only characterise the dominant DNA sequence within an amplicon [80, 86]. Filarial worm coinfections that were identified by metabarcoding typically had one pathogen with a substantially higher read count than the other. This could potentially reflect the predominance of one pathogen within the host, or be a by-product of sampling timepoint, as many filarioid nematodes generate periodical fluctuations of microfilaremia [21, 75]. The superior sensitivity of the metabarcoding assay over the cPCR method may be due to substantial improvements made to the sequencing library preparation protocol. For example, the addition of a NucleoMag bead purification step, utilising a ratio of beads that allowed for exclusion of amplicon products below 200 bp, optimised our method and increased the amount of COI gene reads obtained during a sequencing run. Such simple adjustments reduced wasted sequencing effort on short, non-informative reads (possible PCR artefacts) and likely benefited the metabarcoding assay’s overall sensitivity.

The results of the MKT identified less filarial worm infections than both metabarcoding and cPCR with Sanger sequencing. This method missed 17 *Brugia* spp. and 34 *Dirofilaria* spp. infections detected by the metabarcoding assay i.e., of the total infections for these pathogens 89% and 64% respectively, were only found by metabarcoding. Kappa agreement statistics were defined as poor for *Brugia* spp. and fair for *Dirofilaria* spp. due to discrepancies in samples identified as positive. These results demonstrate serious shortcomings in the relative sensitivity of microscopy-based methods like the MKT that may miss significant numbers of filarial worm infections if employed for epidemiological surveys and clinical diagnosis [21, 22, 75]. Moreover, diagnostic concordance between the MKT and cPCR with Sanger sequencing was also suboptimal, with six *Brugia* spp. and 15 *Dirofilaria* spp. positive samples missed by the MKT. Despite the lower apparent sensitivity of the MKT, this method may still be valuable for use in certain research and clinical contexts given it is much cheaper and quicker to perform than molecular-based methodologies.

Interestingly, one microscopy positive sample for *Dirofilaria* spp. went undetected by both molecular techniques employed by this study. For this sample, only one microfilaria was detected via examination of a stained blood smear implying a very low level of microfilaremia. Given that the MKT utilises 1 ml of whole blood, compared to the 200 µl used for DNA extraction this result may be due to an absence of microfilariae within the 200 µl of blood used for molecular tests or, alternatively, PCR inhibitors carried over from DNA extraction may have been present in this sample, preventing amplification of filarial DNA [87].

The mitochondrial COI gene targeted by our metabarcoding assay poses some minor risks regarding its potential to overinflate species diversity due to the presence of nuclear mitochondrial pseudogenes (NUMTs) present in some filarial worms, particularly those in the genus *Mansonella* [88, 89]. Assays targeting the COI gene of *Mansonella ozzardi* have been shown to occasionally detect such NUMTs, which can be identified by the fact that they generate premature stop codons or frame shift mutations when translated [88, 89]. All COI genes sequenced in this work were identified as being of mitochondrial origin, however when employing this metabarcoding assay, generated sequences should be translated to ensure that they do not form a truncated COI gene or frame shift mutation, indicative of being derived from a NUMT [90]. It is important to note that the significantly longer amplicon generated by our metabarcoding protocol reduces the likelihood of NUMT sequencing as the bioinformatic parameters used by our assay would filter out the majority of sequences that were outside of our target amplicon’s normal range [90].

Through iterative improvements of our metabarcoding protocol it was decided that for simplicity, after the method’s second PCR, cleaned PCR product would be pooled in equal amounts (3 µl per sample), irrespective of DNA concentration. Although equimolar concentrations of PCR product are typically recommended to achieve approximately equal numbers of reads for each sample across a sequencing run, i.e., achieving balanced sequencing effort, this was not possible in the context of our assay as samples had a wide range of DNA concentrations after the secondary PCR. These concentrations were typically correlated with if a sample was filarial worm positive or negative.

Whilst ONT’ flow cells can typically be reused multiple times after flow cell washing with DNase, we did not reuse flow cells for our metabarcoding assay, due to the risk of DNA carry-over between sequencing runs [43]. Given the long 43-hour duration of sequencing chosen for our batch of 96 multiplexed samples, further exploration could be conducted to identify the amount of sequencing time required to maintain a high level of sensitivity to filarial worm infection. Such sequencing durations could likely be substantially reduced given the high read counts attained by our filarial worm positive samples, especially if less than 96 samples are to be processed within one sequencing batch.

## Conclusions

We present the first proof-of-concept study to show how nanopore sequencing can be used to characterise filarial worm species and communities from the blood of mammalian hosts, by sequencing of the COI gene to obtain the metabarcode for this parasite group. The superiority of this assay for detecting filarioids when compared to traditional diagnostic methods, such as the MKT and cPCR with Sanger sequencing, is demonstrated through the larger number of infections detected and improved ability to detect coinfections, which were common throughout our test group. These data are important given the frequent use of traditional diagnostics for epidemiological surveys of filarial worms in both humans and animals, highlighting the importance of future deployment of more refined and sensitive diagnostic techniques for better unravelling of parasite prevalence and transmission dynamics [91–96]. Our herein developed metabarcoding assay could show broad application to the detection of filarial worms from diverse vectors and animal hosts, including humans, analogous to the way 16S and 18S rRNA metabarcoding of bacteria and protozoa has been used to detect vector-borne pathogens from ectoparasites and livestock [97–102]. Previous bacterial metabarcoding methodologies have demonstrated the potential for detection of the common filarioid endosymbiotic bacteria *Wolbachia* spp. to be used as a possible proxy for filarial worm infections in canine hosts [43, 79]. Therefore, combining these 16S rRNA targeting methods with those developed for sequencing the filarioid COI gene may further improve the sensitivity of these protocols and shed more light on the phylogeny, diversity, and speciation of these important endosymbiotic bacteria, with implications for filarial worm control [103–105]. Overall, the metabarcoding assay’s improved ability to detect coinfections means that its employment may be significantly better than conventional diagnostics at elucidating pathogen transmission dynamics, identifying novel animal reservoirs of zoonotic pathogens and better understanding filarial worm ecology.

### Electronic supplementary material

Below is the link to the electronic supplementary material.

Supplementary Material 1

## Data Availability

The GitHub page detailing how our filarial worm database was constructed is available here https://github.com/vetscience/Huggins_NanoCLUST, with our database downloadable from https://melbourne.figshare.com/projects/Huggins_NanoCLUSTdb/160631. All metabarcoding NGS data produced in the present study are available from the NCBI BioProject database BioProjectID: PRJNA923029; BioSampleIDs SAMN32683217 to SAMN32683322, specifically Sequence Read Archive (SRA) accessions SRX19025169 to SRX19025274.

## Abbreviations

CI: Confidence interval
COI: Cytochrome c oxidase subunit 1
cPCR: Conventional polymerase chain reaction
ITS2: Internal transcribed spacer 2
MDA: Mass drug administration
MKT: Modified Knott’s test
NGS: Next-generation sequencing
ONT: Oxford Nanopore Technologies
SL: Sri Lanka
