# Supplementary material for: Development and validation of a long-read metabarcoding platform for the detection of filarial worm pathogens of animals and humans
Source: BMC Microbiol. 2024 Jan 20;24:28. doi: 10.1186/s12866-023-03159-3 (PMC10799534; doi:10.1186/s12866-023-03159-3)
Supplement: Supplementary file 1 — Supplementary Material 1 [file 12866_2023_3159_MOESM1_ESM.docx]

**Additional Information 1:** **DNA sequence of the unique positive control gBlock construct used for filarial worm cytochrome c oxidase subunit I gene (COI)-targeting metabarcoding characterisation.**

The gBlock positive control DNA sequence construct (690 bp) is comprised of COIintF and COIintR primer binding sites (underlined) and a region of the 16S ribosomal RNA gene of *Aliivibrio fischeri*. This construct was synthesised by Integrated DNA Technologies (Iowa, USA).

**5’ –** TGATTGGTGGTTTTGGTAAATTGAACGCTGGCGGCAGGCCTAACACATGCAAGTCGAGCGGAAACGACTTAACTGAACCTTCGGGGAACGTTAAGGGCGTCGAGCGGCGGACGGGTGAGTAATGCCTGGGAATATGCCTTAGTGTGGGGGATAACTATTGGAAACGATAGCTAATACCGCATAATGTCTTCGGACCAAAGAGGGGGACCTTCGGGCCTCTCGCGCTAAGATTAGCCCAGGTGAGATTAGCTAGTTGGTGAGGTAAGAGCTCACCAAGGCGACGATCTCTAGCTGGTCTGAGAGGATGATCAGCCACACTGGAACTGAGACACGGTCCAGACTCCTACGGGAGGCAGCAGTGGGGAATATTGCACAATGGGCGAAAGCCTGATGCAGCCATGCCGCGTGTATGAAGAAGGCCTTCGGGTTGTAAAGTACTTTCAGTAGGGAGGAAGGTGTTGTAGTTAATAGCTGCAGCATTTGACGTTACCTACAGAAGAAGCACCGGCTAACTCCGTGCCAGCAGCCGCGGTAATACGGAGGGTGCGAGCGTTAATCGGAATTACTGGGCGTAAAGCGCATGCAGGTGGTTCATTAAGTCAGATGTGAAAGCCCGGGGCTCAACCTCGGAACCGCATTTGAAACTGGTGAACTAGAGTGCTGTAGAGGGATATTGATACTCGTACTTAT**– 3’**
